# Supplementary material for: Controlling photothermoelectric directional photocurrents in graphene with over 400 GHz bandwidth
Source: Nat Commun. 2024 Aug 27;15:7351. doi: 10.1038/s41467-024-51599-w (PMC11347599; doi:10.1038/s41467-024-51599-w)
Supplement: Supplementary file 1 — Supplementary Information [file 41467_2024_51599_MOESM1_ESM.pdf]

Supplementary Information for

Controlling Photothermoelectric Directional Photocurrents in Graphene with over 400 GHz Bandwidth

Stefan M. Koepfli[1]*, Michael Baumann[1], Robin Gadola[1], Shadi Nashashibi[1], Yesim Koyaz[1,2], Daniel Rieben[1], Arif Can Güngör[1], Michael Doderer[1], Killian Keller[1], Yuriy Fedoryshyn[1], Juerg Leuthold[1]*

1. *ETH Zurich, Institute of Electromagnetic Fields (IEF), 8092 Zurich, Switzerland*
2. *Now with EPFL, Photonic Systems Laboratory (PHOSL), 1015 Lausanne, Switzerland.*

**corresponding email: stefan.koepfli@ief.ee.ethz.ch, juerg.leuthold@ief.ee.ethz.ch*

The PDF file includes:

Supplementary Notes 1-9
Supplementary Figures 1-18
Supplementary Table 1

Supplementary Note 1: Seebeck Modelling

To model the Seebeck coefficient in the channel, we use the Mott formula as provided in Ref.[1]

$$S(\mu,\varphi) = \frac{2\pi k_B^2 T_e}{3\hbar^2 v_F^2} \times \frac{\mu\,\mu_c}{\sigma_{min} + \frac{e}{\pi\hbar^2 v_F^2}\;\mu\,\mu_c^2}. \quad \text{Supplementary Equation (1)}$$

We make the simplified assumption that the electron temperature is constant: $T_e = 300\,K$. We further assume a Fermi velocity of $v_F = 10^6\,m/s$. Apart from the constants (k_B Boltzmann constant, $\hbar$ reduced Planck constant, e elementary charge) this leaves the graphene carrier mobility μ, the minimum conductivity in the Dirac point σ_{min} and the chemical potential μ_c. Here, we approximate the chemical potential μ_c with the Fermi level at $0\,K$, i.e. we set $\mu_C \approx W_F$.

Firstly, to model the carrier density of graphene within the channel we use a simplified approach from ref[2]. The carrier concentration is described by

$$\Delta n(W_F) = \int_{-\infty}^{W_F} f(W')DOS(W')dW'' \sim sgn(W_F)\frac{1}{\pi}\left(\frac{W_F}{hv_F}\right)^2, \quad \text{Supplementary Equation (2)}$$

where $f(W)$ represents the Fermi Dirac distribution and $DOS(W)$ the density of states. We have assumed $T = 0\,K$ which reduces the Fermi Dirac distribution after the integration to a signum function and leaves the linear density of states $DOS(W) = \frac{|W|}{\pi h^2 v_F^2}$ that describes the typical graphene cone-like band structure. To solve for the electrostatic potential change, we solve a partial differential equation (PDE) in the form of a Poisson equation

$$\nabla(\varepsilon_r \nabla\varphi) = -\frac{\rho(\varphi)}{\varepsilon_0}. \quad \text{Supplementary Equation (3)}$$

Here, the area charge density is $\rho(\varphi) = \Delta n(W_F)e$, where W_F is the Fermi level, i.e. the level at $0\,K$ shifted by $e\varphi$. We describe the carrier concentration as $n^2 = n_0^2 + \Delta n^2$, where n_0 the residual charge carrier concentration and Δn the induced carrier concentration. In this theoretical model, we set $n_0 = 0$ for graphene and the above equation simplifies to $\rho(\varphi) = n(W_F)\,e$. Combining Eq. S2 and S3 one obtains the charge density

$$\frac{\rho}{\varepsilon_0} = \frac{-e}{\varepsilon_0} sgn(\varphi)\frac{1}{\pi}\frac{(-e\varphi)^2}{(hv_F)^2}, \quad \text{Supplementary Equation (4)}$$

which we use as a Neumann-type boundary condition (BC) in a general 3D PDE solver. The metallic resonators form a fixed potential Dirichlet BC and the gate electrode forms a controllable Dirichlet BC directly equal to the applied gate voltage V_G. Solving for the potential φ as a function of position and applied gate voltage V_G, one arrives at the spatial potential distribution $W_F(x, y, V_G)$. The results are provided in Supplementary Fig. 1.

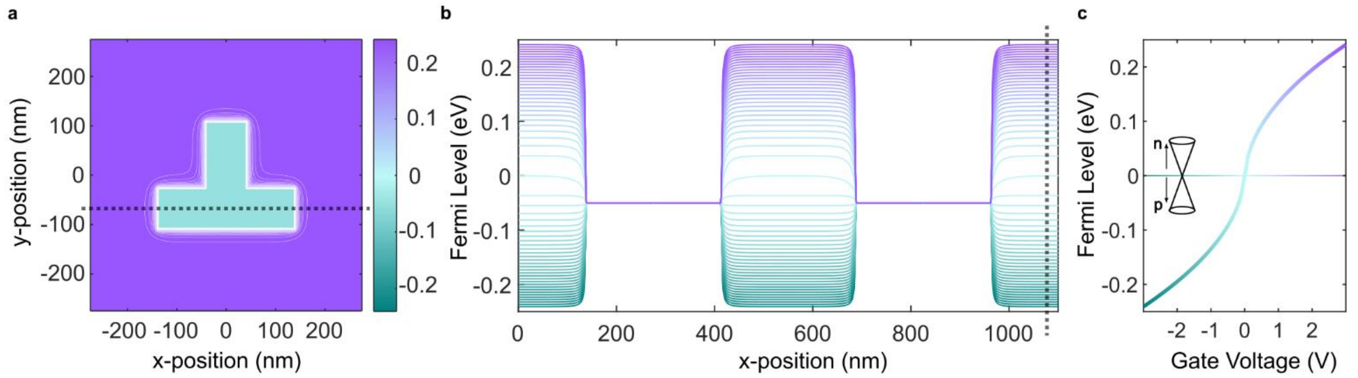

Supplementary Fig. 1: **Graphene potential simulation**.
a Ferm level W_F of a graphene sheet for a single unit cell of the metamaterial with 0 V applied gate. The metallic resonator dopes the graphene which also extends into the resonator. **b** Cross-section of the Fermi level along the black dotted line in **a** plotted across two unit cells. The different traces correspond to gate voltages V_G from -3 V to 3 V in steps of 0.1 V. **c** Fermi level in the graphene away from the resonator (black dotted line in **b**) linking the gate voltage to the induced potential shift.

Next, we extract σ_{min} and μ directly from the resistance measurements provided in Fig. 2b. We follow a similar approach to ref[3]. We first convert the resistance to the conductivity (Supplementary Fig. 2a). The Dirac point conductivity corresponds to $\sigma_{min} = 0.172\ mS$. To calculate the mobility, we relate the gate voltage V_G to the Fermi level shift using the simulation results presented in Supplementary Fig. 1c. Converting the potential to the carrier concentration by $\Delta n = \frac{1}{\pi}\left(\frac{W_F}{\hbar v_F}\right)^2$ and plotting it against the measured conductivity on a double log scale (Supplementary Fig. 2b) allows to extract the residual carrier concentration $n_0 \approx 5 * 10^{11}\ \mathrm{cm}^{-2}$. With this, the carrier concentration $n^2 = n_0^2 + \Delta n^2$ is now linked to the gate voltage (Supplementary Fig. 2c) which allows to relate the measured conductivity to the carrier concentration (Fig S2d).

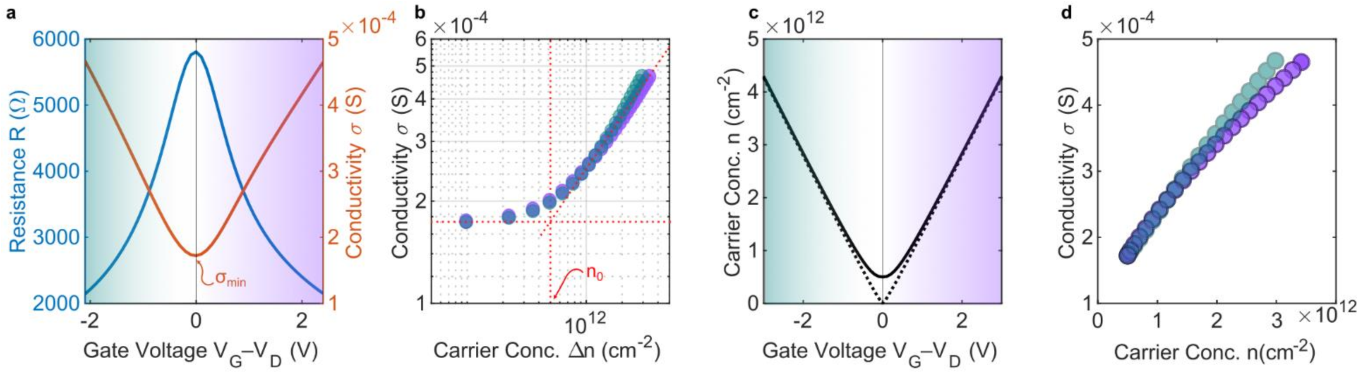

Supplementary Fig. 2: **Graphene property calculations**.
a Measured device resistance (left axis) and resulting conductivity (right axis) as a function of gate voltage. The minimum conductivity σ_{min} is extracted from the Dirac point conductivity. **b** Measured conductivity as a function of calculated carrier concentration shift Δn on a double logarithmic scale. The cross-section of the linear slope for large doping values and the level of the minimum conductivity are used to estimate the residual carrier concentration n_0. **c** Carrier concentration n as a function of gate voltage. The dotted line corresponds to the ideal case with $n_0 = 0$, whereas the solid line takes into account the non-zero n_0. **d** Using **a** and **c** to show the measured conductivity as a function of carrier concentration n. The slope corresponds to the graphene carrier mobility μ.

The mobility is then calculated with:

$$\mu = \frac{1}{e}\frac{\Delta\sigma}{\Delta n}$$

and is found to be $\mu_0 \approx 900\ cm^2/Vs$ close to the Dirac point.

Using all extracted values, the resulting Seebeck coefficient as a function of gate voltage is given Supplementary Fig. 3a. We also provide example curves in Supplementary Fig. 3b on how the Seebeck coefficient would be influenced if the carrier mobility would be improved (1’000, 2’000, 5’000, 10’000 and 20’000 cm^2/Vs) Furthermore, the maximum Seebeck coefficient as a function of mobility and minimum conductivity is calculated in Supplementary Fig. 3c. Higher quality graphene (low σ_{min}, high μ) leads to an improved Seebeck coefficient whereas poor quality graphene (high σ_{min}, low μ) leads to a low value. The red square marks our values. We note that for this value the contact resistance is not removed.

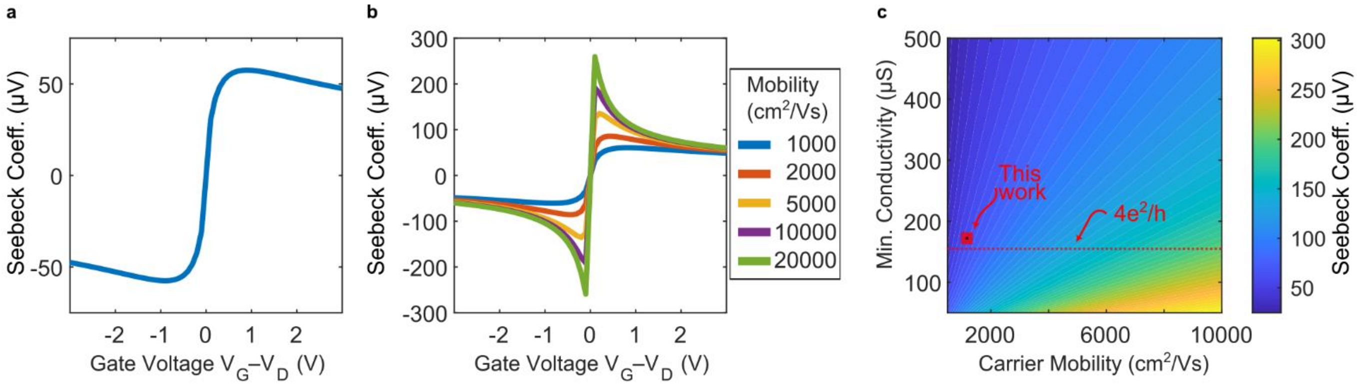

Supplementary Fig. 3: **Resulting Seebeck Coefficient.**
a Seebeck coefficient as function of gate voltage (V_G–V_D, where V_D is the Dirac voltage) for the extracted device parameters. **b** Calculated Seebeck coefficient for higher carrier mobility. **c** Seebeck coefficient as a function of carrier mobility and minimum conductivity. The red square marks the values from **a**.

Supplementary Note 2: Orientation of Resonators

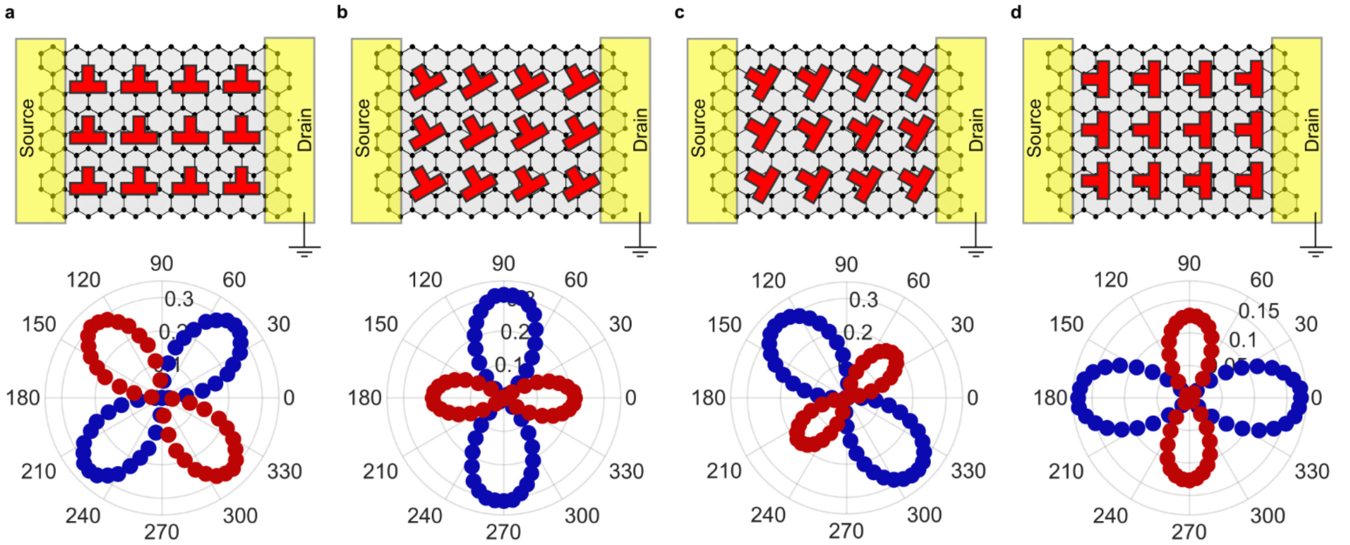

Supplementary Fig. 4: **Polarization dependent photoresponse of resonator orientations.**
a-d Schematic and corresponding measured photoresponse as a function of polarization for resonator orientations of **a** 0°, **b** 30°, **c** 60° and **d** 90°.

Supplementary Fig. 4 shows further resonator orientations in addition to the two resonator orientations provided in the main text: **a** 0°, **b** 30°, **c** 60° and **d** 90°. Rotating the resonators is not equivalent to a rotation of the polarization, as the source-drain orientation defines the projection direction in which the photocurrent is sampled. Each 30° counter-clockwise rotation of the resonator leads to a 45° counter-clockwise (135° clockwise) rotation of the polarization lobes.

The current direction can be described with the induced driving forces as discussed in the main text. The cross-sectional view picture introduced in Fig. 3f of the main text is, however, not sufficient to capture arbitrary rotations of the resonators.

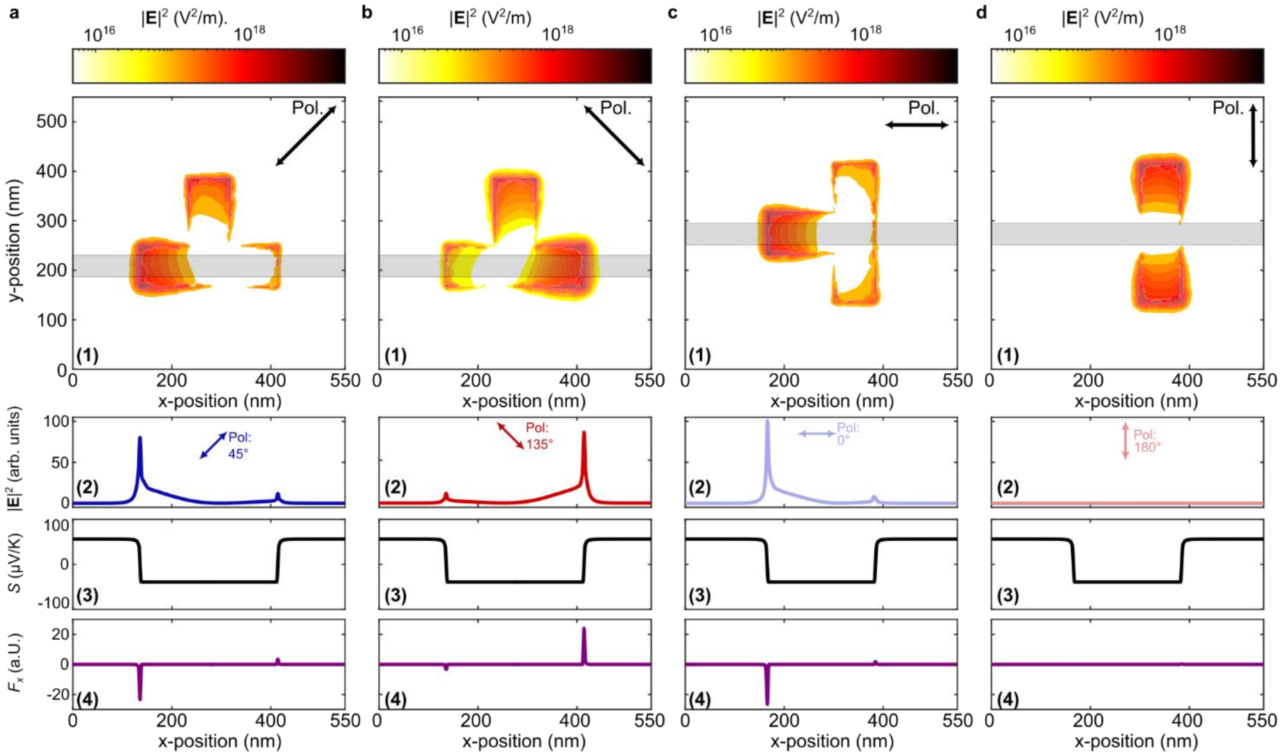

Supplementary Fig. 5: **Limitations of cross-sectional view.**
Driving field calculations F_x by using the electric field intensity $|\mathbf{E}|^2$ and Seebeck coeff. S in a cross-sectional view for four different cases: **a** 0° resonator orientation, 45° polarization. **b** 0° resonator orientation, 135° polarization. **c** 90° resonator orientation, 0° polarization. **d** 90° resonator orientation, 90° polarization. Case **d** predicts 0 current along the x-direction not capturing the device behavior. The gray bar indicates the used cross-section.

The limitation is further visualized in Supplementary Fig. 5. Four cases are presented: Supplementary Fig. 5a,b present the 0° resonator orientation (i.e., baseline orientation) under 45° and 135° polarization excitation. Supplementary Fig. 5c,d present the 90° resonator orientation (i.e., crossbar orientation) for 0° and 90° polarization excitation. For each case, (1) represents the simulated optical field by showing the square of the electric field in the graphene layer. The mean value $\overline{|E|^2}$ for a certain width (marked by a gray area) is extracted along the x-coordinate for each case and given in the cross-sectional view (2). The calculated Seebeck coefficient profile for one fixed gate voltage is provided in panel (3). The resulting force along the x-direction is calculated by $F_x(x) = a * dS(x)/dx * |E(x)|^2$, where a is used as a scaling factor. Panel (4) represents the force for each case Supplementary Fig. 5a through Supplementary Fig. 5d. Supplementary Fig. 5a,b show the photoresponse with its sign and also show a response of equal magnitude. Supplementary Fig. 5c can describe the 0° response, however, Supplementary Fig. 5d shows an F_x close to zero. The measured response, however, is clearly non-zero (Supplementary Fig. 4d). Therefore, the simple model can not fully describe the absorption and full hydrodynamic electron flow models as used in refs[4,5] are needed. They provide a more accurate view on the induced direction of the currents.

Supplementary Note 3: Electron current flow simulations

Following the introduced model in refs[4,5], the electron flow in the unit cell is described with the steady-state Navier-Stokes equation

$$-\gamma\vec{u}(x,y) - (\vec{u}(x,y)\cdot\nabla)\vec{u}(x,y) + \nu\nabla^2\vec{u}(x,y) - \frac{1}{n}\nabla\phi(x,y) = -\vec{F}(x,y).$$

where $\vec{u}$ is the velocity of the carriers, γ is the carrier-carrier scattering rate with $\gamma^{-1} \approx 80\text{fs}$ (see [6]), ν is the dynamic viscosity which can be modelled as $\nu = \frac{1}{2}v_F^2\gamma^{-1}$ (see [6]) with the Fermi velocity that can be approximated by $v_F \approx 10^6\, {}^{m}/_{S}$, the graphene carrier density n can be estimated to be $n \lesssim 10^{11}\, cm^{-2}$ (from the above calculations). Lastly, the force can be calculated with

$$\vec{F}(x,y) = a * \nabla S(x,y) * |E(x,y)|^2,$$

where $a = 10$ is a scaling factor. The Seebeck coefficient S is calculated as described in *Supplementary Note 1*, where the electric field $E(x,y)$ follows from optical simulations (see Methods). The outer boundaries are chosen as periodic boundaries. The simulation flow is depicted in Supplementary Fig. 6 for the 90° polarization.

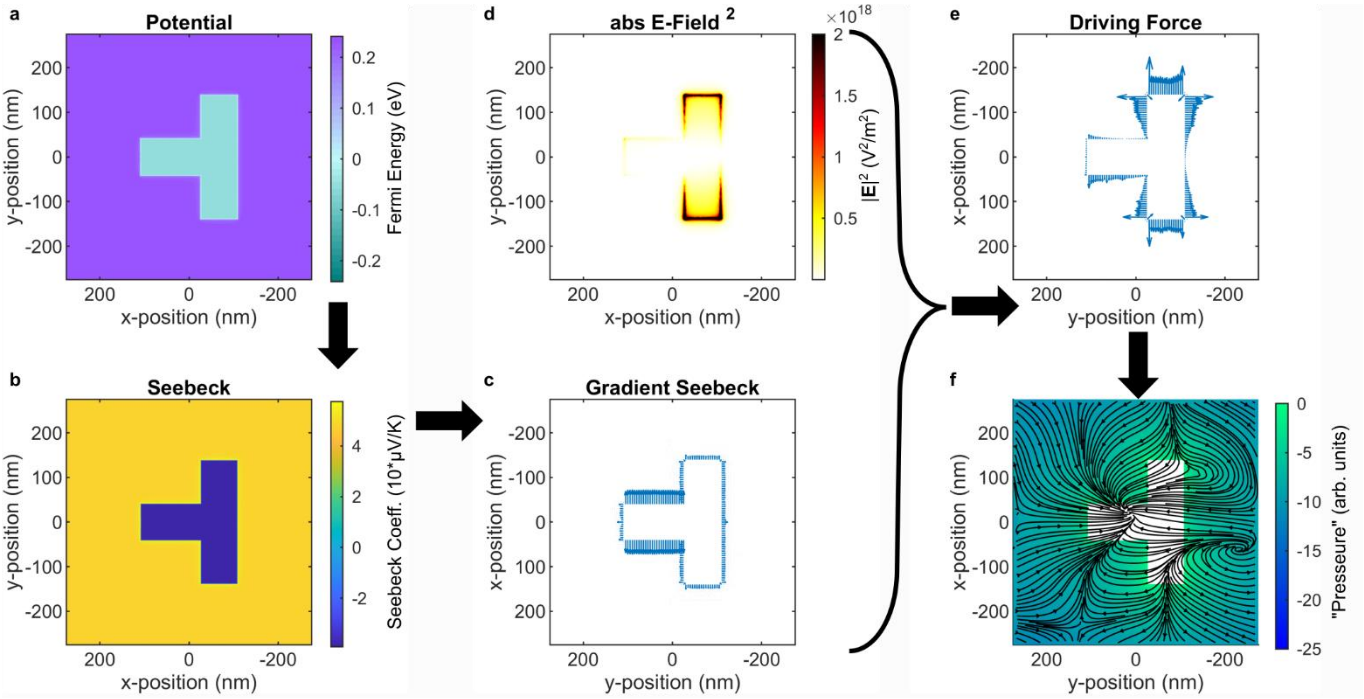

Supplementary Fig. 6: **Electron flow simulation chain.**
a The electrostatic potential of the metamaterial unit cell is simulated by considering the contact doping of the resonator and the applied gate voltage as described in Supplementary Note 1. **b** From the potential distribution, the Seebeck coefficient is calculated. **c** The gradient of the Seebeck coefficient is calculated and multiplied together with **d** the simulated electric field response of the metamaterial unit cell. **e** Calculated driving force which is fed into the hydrodynamic flow simulations to calculate **f** the carrier flow.

Even though the electric field and driving forces have symmetry axis (Supplementary Fig. 6b,e), a net flow towards the left is captured in this model. The crossbar of the resonator acts as sink due to the induced potential landscape: Carriers pushed by the driving force to the right are free to move, whereas carriers pushed to the left are being pulled towards the lower potential. We note that the model in this state is describing the phenomenon of the directional photocurrents but does not provide a direct measure of the photoresponsivity.

We further provide electric field simulations for polarizations from 90° to 270° in steps of 10° in Supplementary Fig. 7. The field behavior intuitively shows how the directional carrier flow comes to

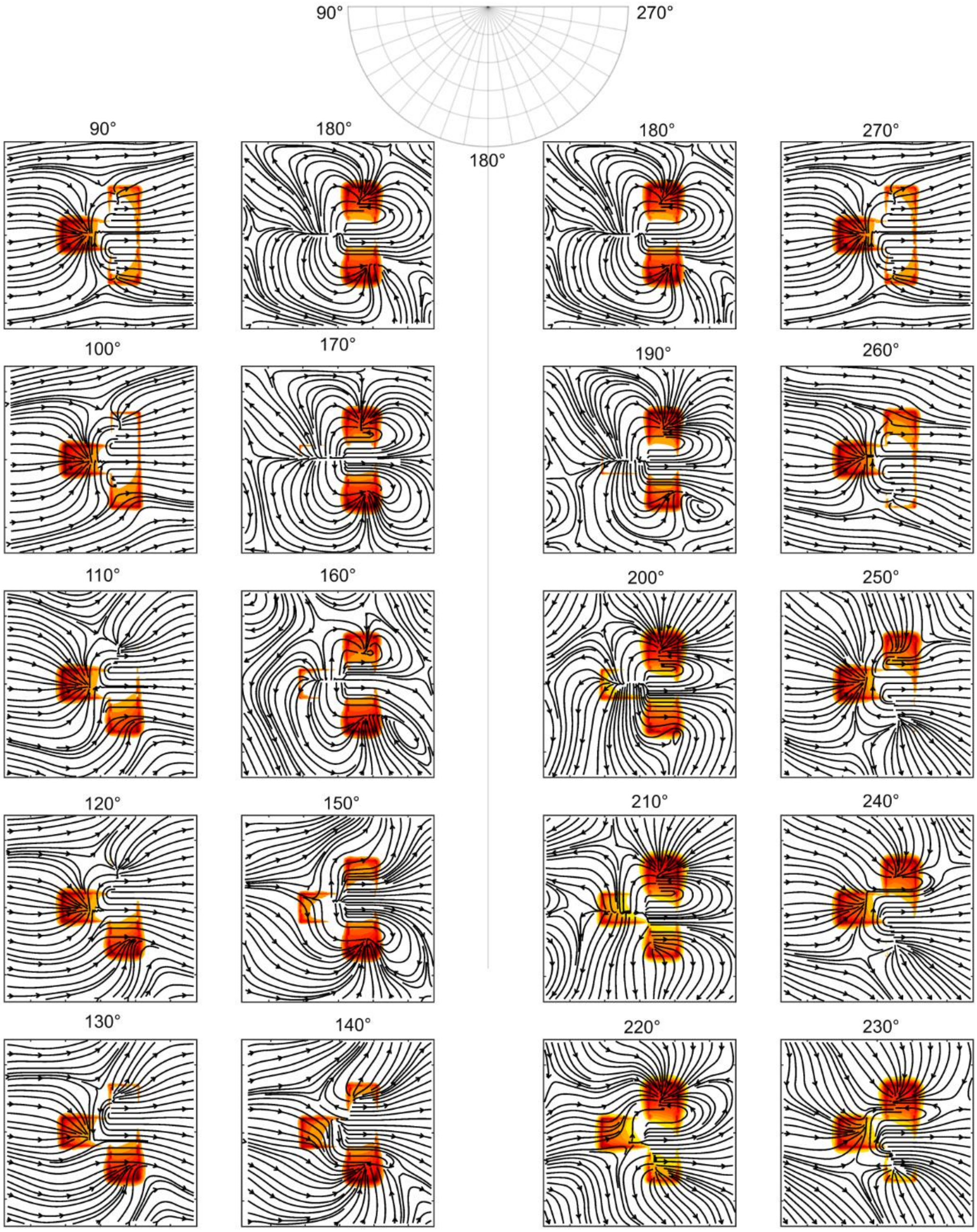

Supplementary Fig. 7: **Polarization dependent field and induced carrier flow behavior.**
Metamaterial resonators under different polarized excitations and the resulting $|\mathbf{E}|$ pattern (heatmap) and induced carrier flow. Rotating the polarization from 90° (see polar grid for orientation definition) to larger angles results in an asymmetric field distribution which starts to twist the flow upwards until it reaches 180° where the flow direction is weaker and reversed in comparison to the 90° case. Further turning the polarization beyond 180° twists the flow downwards until it rotates back to the initial flow direction for the 90° / 270° polarization.

Supplementary Note 4: Metamaterial Perfect Absorber Design

Supplementary Fig. 8a shows a schematic 3D unit cell of the metamaterial perfect absorber layer stack. One of the most crucial parameters to fulfill the impedance matching condition and achieve perfect absorption is the spacer thickness. Fig. 8b shows the simulated absorption spectrum as function of spacer thickness. Several spacer thicknesses meet the impedance matching condition, leading to theoretically perfect absorption of 100%.

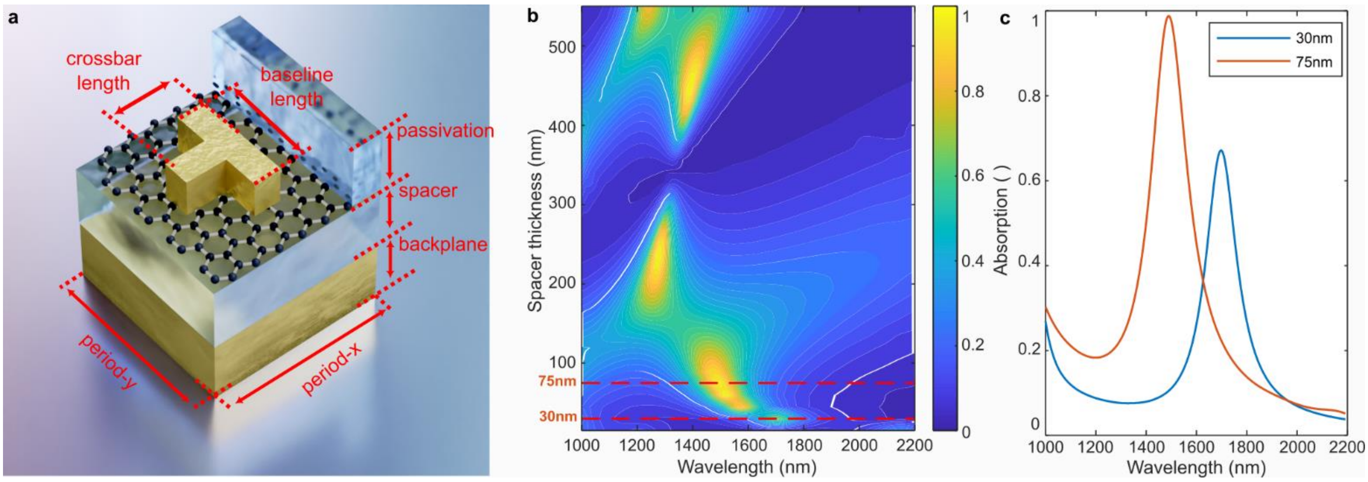

Supplementary Fig. 8: **Metamaterial perfect absorber design – spacer thickness.**
a Schematic 3D view of a unit cell of the metamaterial perfect absorber layer stack with the labelled parameters. **b** Simulated absorption spectrum as a function of spacer thickness. Several spacer thicknesses allow to reach high to perfect absorption (yellow spots). The polarization is oriented along the crossbar. **c** Example absorption spectra for 30 nm spacer thickness and 75 nm spacer thickness.

Supplementary Fig. 8c shows the spectra of the two marked spacer thicknesses (30 nm and 75 nm). Clearly, perfect absorption is reached with the 75 nm case. Nevertheless, the presented structures were fabricated with 35 nm of spacer thickness due to the trade-off in required gate voltages. Scaling the spacer layer thickness immediately scales the required gate bias voltage. To operate in the low voltage regime, using the first resonance is beneficial, but comes at the cost of reduced absorption.

Fig. S9 provides more details on the presented simulations from Fig. 2(a) on the tunability of the absorption by means of the resonator geometry. Thereby, Supplementary Fig. 9b,c and Supplementary Fig. 9e,f expand the analysis by showing the absorption for polarized light. From these plots and the electric field pattern of Fig. 1c,d in the main text, the absorption bands be associated to the resonator structure dimensions.

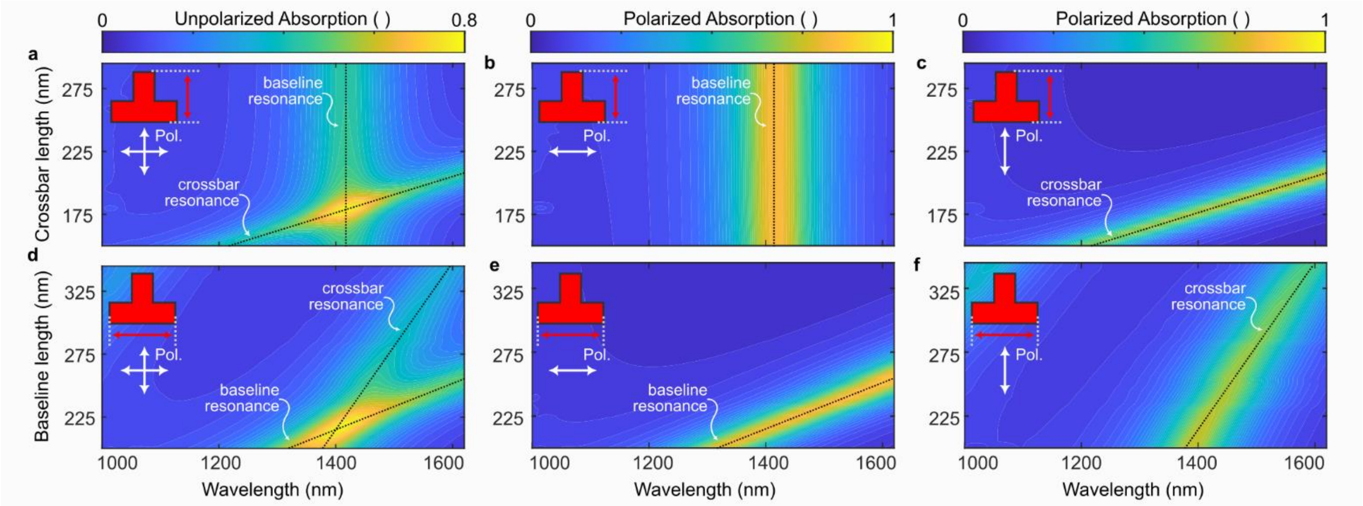

Supplementary Fig. 9: **Metamaterial optical absorption simulation for different polarizations.**
a,d Simulated unpolarized absorption of the structure as function of wavelength and **a** crossbar- and **d** baseline length variation as presented in the main text Fig. 2. **b,e** Simulated polarized absorption for both the crossbar- and baseline length sweeps. The polarization is aligned along the baseline of the resonator (indicated by the white arrow). **c,f** Simulated polarized absorption for both length sweeps with the polarization aligned along the crossbar of the resonator (indicated by the white arrow).

Supplementary Note 5: Three port devices

The directionality of the current can further be used to detect the linear polarization state of the light as demonstrated by Wei et al.[4,7] The metamaterial resonator with a three-arm configuration (here referred to as "propeller") can be incorporated in the same metamaterial perfect absorber layer stack to enhance the absorption. Supplementary Fig. 10a shows a schematic unit cell of a propeller resonator in the metal-insulator-graphene-insulator layer stack. We demonstrate the flexibility of the design by simulating the absorption spectrum of the metamaterial for different propeller lengths, see Supplementary Fig. 10b. From this figure one can see that the structure reaches high total absorption >80% and the peak position can be controlled throughout the whole NIR spectral regime simply by changing one geometrical parameter. Supplementary Fig. 9c shows the corresponding measured absorption spectra. A close match between simulation and measurements is observed, considering a size difference of 14 nm between designed and fabricated structures, which is within fabrication tolerances.

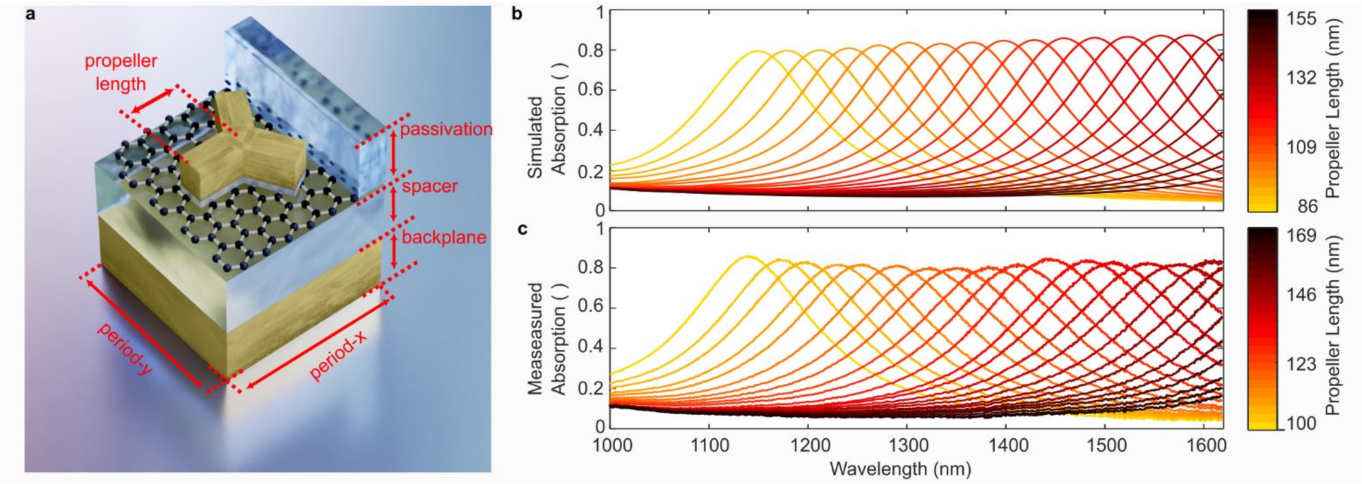

Supplementary Fig. 10: **Three arm resonator metamaterial absorber**.
a Schematic unit cell of the three arm resonator («propeller») unit cell. The stack again consists of a gold backplane, an aluminium oxide spacer layer, a monolayer graphene and the resonator. The full stack is encapsulated with an additional aluminium oxide spacer layer serving as passivation. The overall layer stack is identical to the presented T-resonators in the main text. **b** Simulated optical absorption in the NIR regime for varying propeller lengths (see labelling in **a**). **c** Measured optical absorption for varying propeller lengths. The simulation and measurement results match well if a deviation in propeller length of $\Delta \approx 14\ \mathrm{nm}$ is assumed, which is well within fabrication tolerances.

Fabricated active structures with three contact pads are shown in Supplementary Fig. 11a-c. The graphene sheet is etched into a triangular shape and contacted by three separate contact pads. Each contact is connected to an ammeter to measure the current at each pad as visualized in the schematic in Supplementary Fig. 11d. The measured photoresponse for each channel as a function of polarization angle is provided in Supplementary Fig. 11e (channel 1), Supplementary Fig. 11f (channel 2) and Supplementary Fig. 11g (channel 3). The current in each channel follows a sinus-like shape with a periodicity of 180° - matching with expectation for linear polarized light. Each channel has a 60° polarization shift to the next one. Photocurrent sign switch and the polarization dependence as a function of resonator orientation with respect to the contact pads again highlight the directionality of the current. As previously demonstrated[7], the three electrode design offers a calibration-free linear polarization state detection as the system fulfils Kirchhoff's circuit law and thereby offers two independent equations to determine the polarization angle and incident optical power.

Rotating the resonators by 60° leads to a sign switch in photocurrent as depicted in Supplementary Fig. 11h-k. Polarizations aligned to one of the three arms (0°, 60°, 120°) are now pointing away from the channels rather than pointing to the channels as in Supplementary Fig. 11d. The 60° rotation is essentially a mirroring of the resonator with respect to each channel, thereby inverting the sign of the photocurrent.

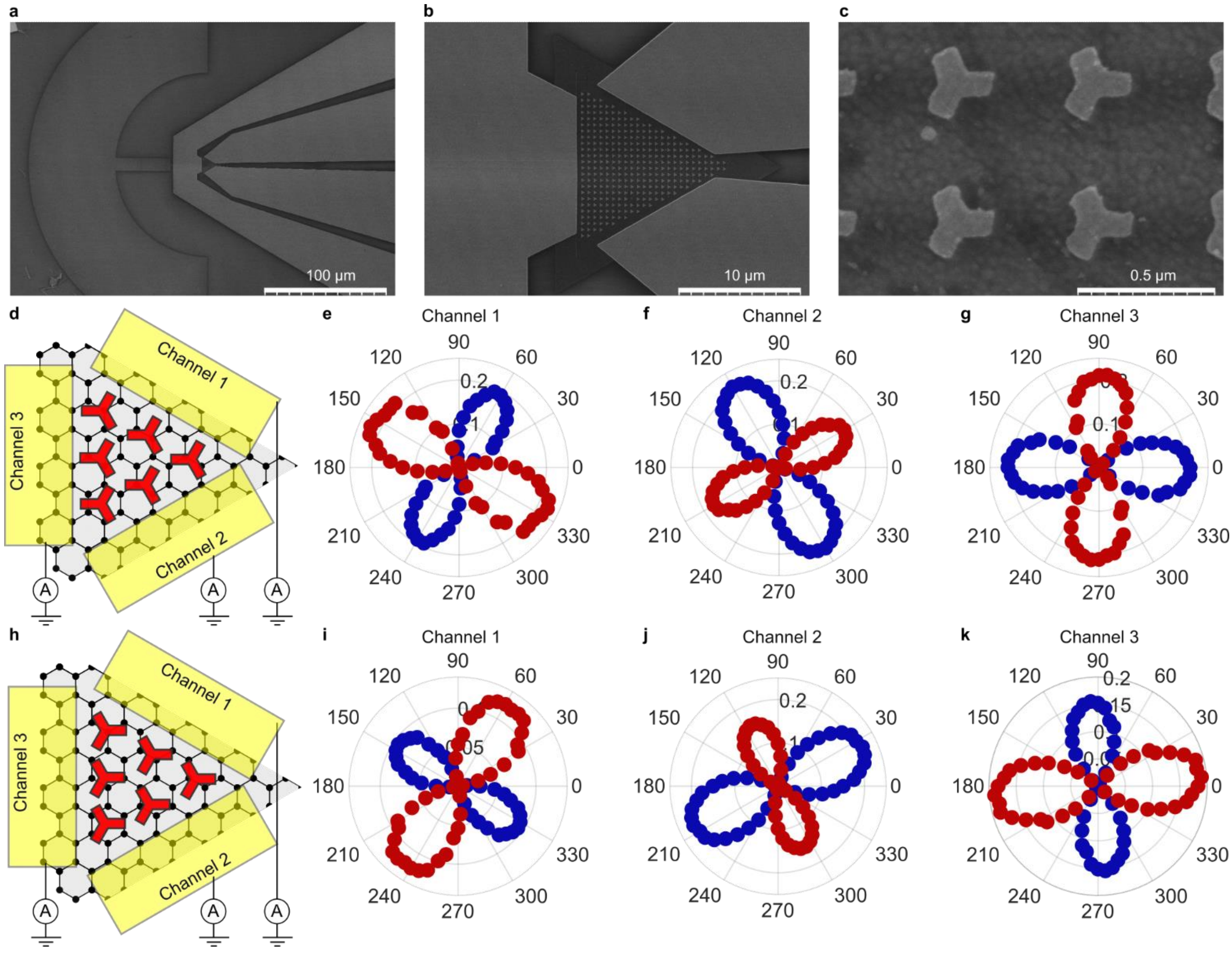

Supplementary Fig. 11: **Three-port propeller metamaterial photodetectors.**
a-**c** Fabricated active three-port devices in various magnifications (**a** 100 µm, **b** 10 µm, **c** 500 nm). The device has three contact channels (1-3) and an additional gate electrode as labelled. Resonators are optimized for operation at 1550 nm. **d** Schematic of the device and the electrical measurement configuration. **e**-**g** Measured photoresponse as a function of polarization angle for **e** channel 1, **f** channel 2 and **g** channel 3. Each channel has a 60° offset with respect to the next channel. **h** Schematic of a device with the resonators rotated by 60° with respect to the device presented in **d**. **i**-**k** Measured photoresponse as a function of polarization angle for the device with rotated resonators for **i** channel 1, **j** channel 2 and **k** channel 3. The three channels now show a sign flipped response (i.e. a polarization shift of 90°) compared to the other orientation.

The architecture can be further optimized for higher absorption and can be further tuned into the mid- infrared by making changes to the layer stack. Figure S12 shows simulated absorption measurements for a spacer thickness of $s = 35\,nm$, a resonator thickness of $t = 33\,nm$ and no passivation layer (see Supplementary Fig. 10a for the meaning of the dimensions). Again, simply by tuning the propeller length the resonance can be kept high (>90% absorption) while moving the peak position through a large than 1500 nm spectral window.

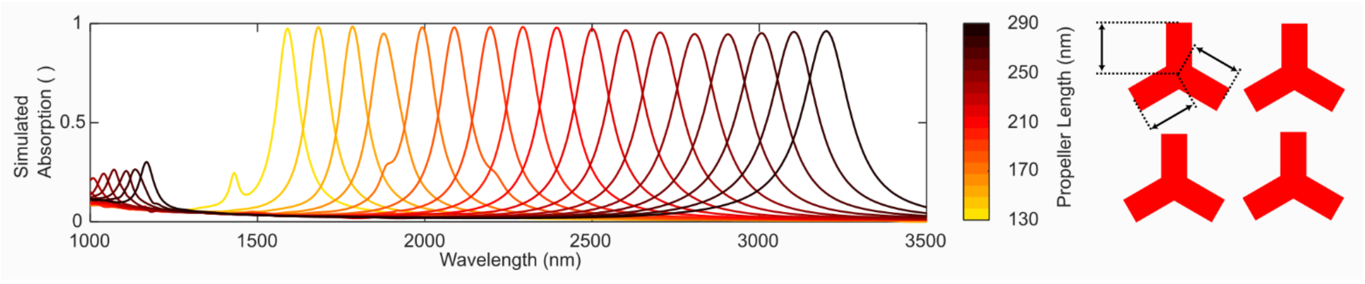

Supplementary Fig. 12: **Simulated MIR regime absorption.**
Simulated absorption of an optimized propeller resonator structure with simulated adaptability in the NIR and MIR regime showing adaptability of the peak position by more than 1500 nm while keeping a high total absorption >90%.

Supplementary Note 6: Four port device crosstalk

Supplementary Fig. 13 represents the data from main text Fig. 6f,g on a logarithmic scale to visualize the crosstalk between the two channels. Supplementary Fig. 13a,b correspond to the wavelength multiplexing case using 1310 nm and 1490 nm lasers and recording channel 1 (a) and channel 2 (b). The cross talk is in the range of $1.5 - 5.2\%$. Supplementary Fig. 13c,d corresponds to the polarization multiplexing case using two 1490 nm lasers. The crosstalk in this case is in the range of $0.5 - 5.1\%$. Active polarization control could further improve the crosstalk.

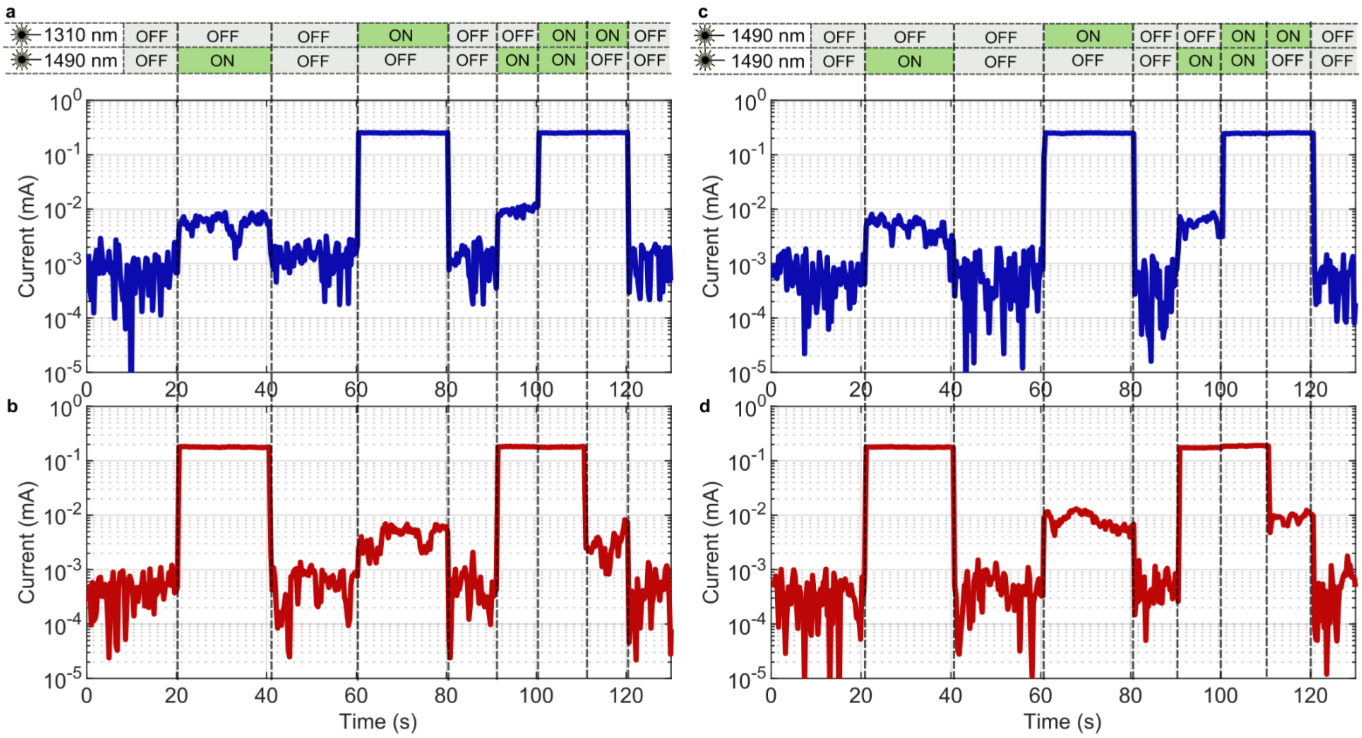

Supplementary Fig. 13: **Multiplexing time traces on a semi-logarithmic scale.**
a-b Time sequence of both channels for alternating and combined illumination under 1310 and 1490 nm from a single single mode fiber visualizing the wavelength multiplexing capabilities. **c-d** Time sequence of both channels for alternating and combined illumination for two differently polarized 1490 nm sources visualizing the polarization multiplexing capabilities.

Supplementary Note 7: NIR and MIR dimension comparison

Supplementary Fig. 14 provides a comparison of a resonator optimized (a) for MIR operation with a close to perfect absorption at 4750 nm and (b) a resonator in the NIR with a roughly 1/5 unit cell size. The electric field distribution as described in the main text Fig. 1c-d is represented for the MIR case in Supplementary Fig. 14c-d. The same lobe pattern is encountered as in the NIR case; however, the field maxima are much more separated. The larger unit cell and resonators thereby distribute the electric field much more than the NIR resonators. This can have beneficial properties on the induced electron flow as not only the electric field term $|E(x,y)|^2$ is modified in the driving force, but also the potential landscape which influences the Seebeck coefficient gradient $\nabla S(x,y)$ and the flow of the carriers.

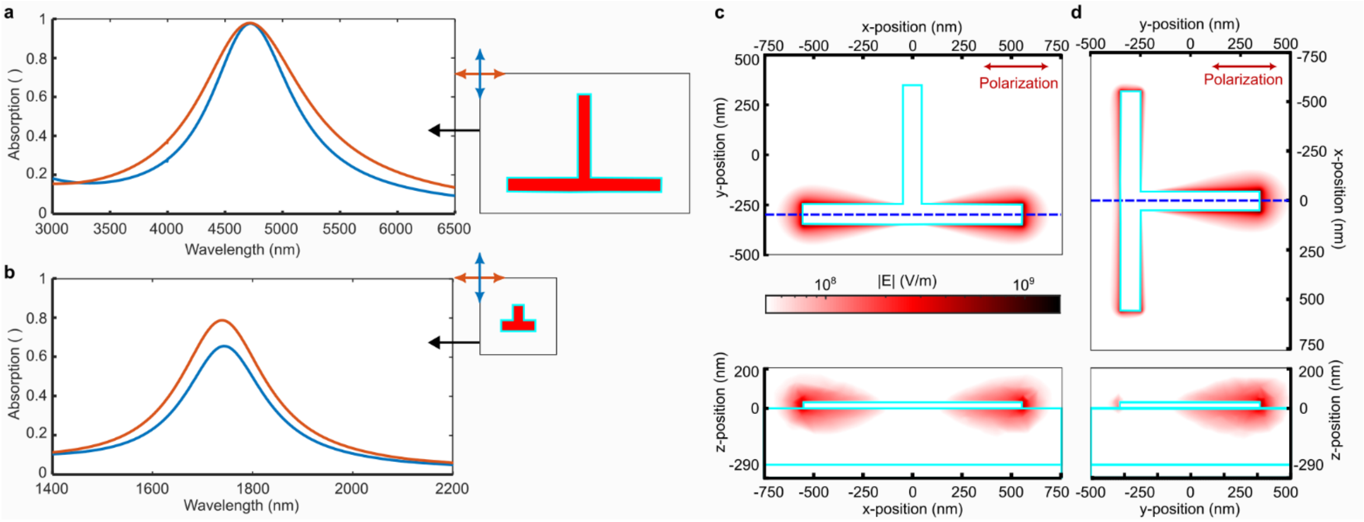

Supplementary Fig. 14: ***Comparison of MIR and NIR optimized resonators.***
a T-resonator metamaterial structure optimized for operation in the MIR range with an absorption peak at 4750 nm. The two curves represent the two different linear polarizations. The schematic on the left visualizes the unit cell of the structure (1000 nm x 1500 nm). **b** Absorption spectrum of a T-resonator metamaterial as presented in the main text. The schematic unit cell is to scale to the larger one presented in **a**, as it only measures 550 nm x 550 nm. **c,d** Optical electric field response of MIR structure under plane wave illumination with the polarizations as indicated. The bottom panel shows the cross-section along the dark blue dashed line in the top panel.

Supplementary Note 8: Discussion on responsivity

At this point, what is still open is the performance in terms of responsivity. The presented devices in this work have responsivities of ~1.5 V/W or ~400-500 µA/W, see also Supplementary Fig. 15.

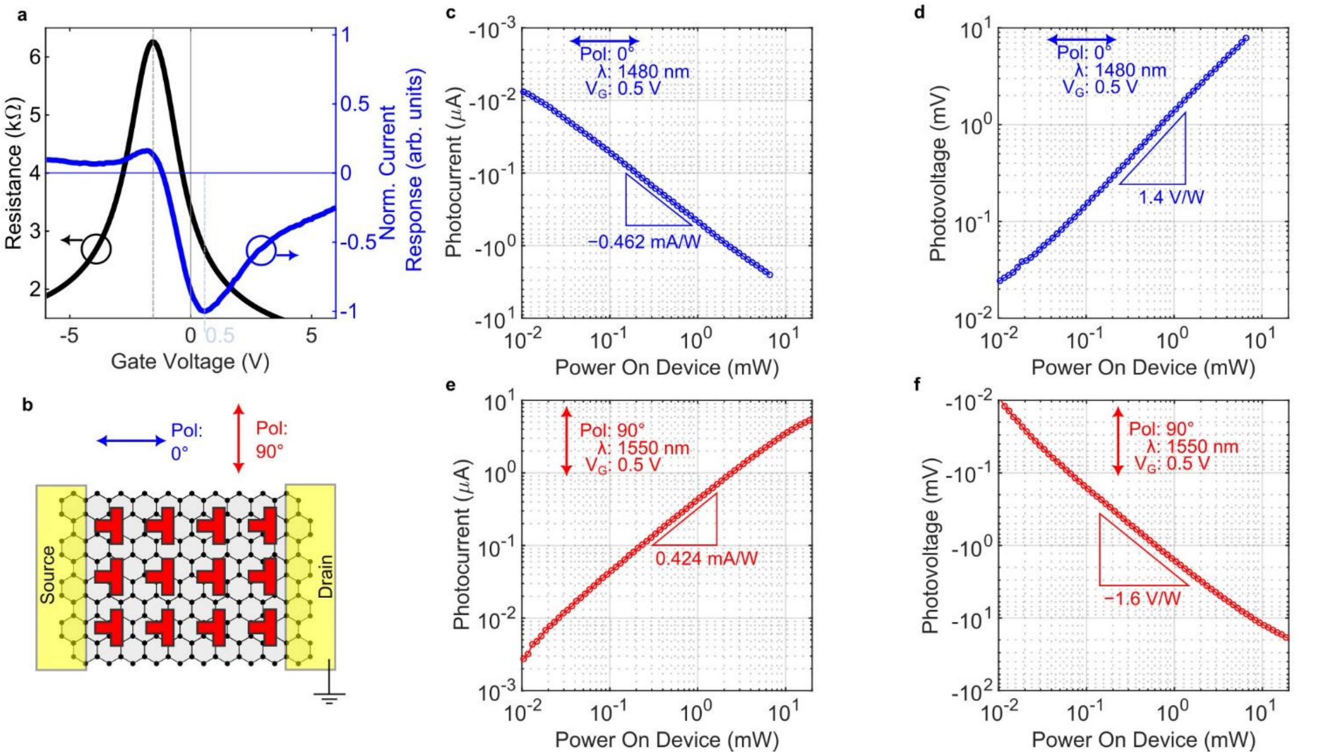

Supplementary Fig. 15: **Voltage and current responsivity and linearity.**
a Device resistance and normalized photocurrent response as function of gate voltage. The maximum current responsivity is found at a gate voltage of 0.5 V where the device resistance is 2.75 kΩ. **b** Resonator orientation of the specified device and the polarization orientation convention used (blue – 0°, red – 90°). **c,d** Photoresponse as function of optical input power for the 0° polarization orientation and a wavelength of 1480 nm. The corresponding responsivities are extracted from the slope as **c** $\mathcal{R}_\mathrm{I} = -0.462\,\mathrm{mA/W}$ and **d** $\mathcal{R}_\mathrm{V} = 1.4\,\mathrm{V/W}$. **e,f** Photoresponse as function of optical input power for the 90° polarization orientation and a wavelength of 1550 nm. The current and voltage responsivities are e $\mathcal{R}_\mathrm{I} = 0.424\,\mathrm{mA/W}$ and **d** $\mathcal{R}_\mathrm{V} = -1.6\,\mathrm{V/W}$. The devices have been illuminated with a lensed fiber (focus spot ~5 µm) to ensure that all light is focused to the active area of the device. A loss of linearity in the 5-10 mW regime is observed.

These responsivity values are considerably lower than the 27 V/W or 36.3 mA/W of the first demonstration[7]. However, the responsivity of the device in this paper can be increased by quite a few measures.

- Moving from high quality mechanically exfoliated few layer graphene to CVD grown polycrystalline monolayer graphene can explain a large fraction of the deficit.
    1) The lower quality, polycrystalline nature leads to more non-uniform potential landscape due to charge puddles[8]. These will lead to perturbations in the long range induced current. However, quantifying the perturbations effects remains challenging.
    2) The typically lower mobility of CVD grown graphene will directly influence the Seebeck coefficient. This would explain a factor $2x$ to $3x$.
    3) A higher layer number will directly scale the absorption in graphene. Even though our device architecture with a metallic backplane leads to a considerable total absorption increase, transitioning to a ~8-layer flakes (such as in Ref. [7]) is likely to lead to another factor $4x$ higher responsivity.
- In this work all experiments have been performed in the near infrared between 1.3 and 1.5 µm. The experiments in Ref. [7] have been performed at 4 µm. While shrinking the resonators successfully works to blue shift the absorption, it does not directly scale with the induced electron flow due to simultaneous changes in the potential landscape (see Supporting Information). Our estimate results indicate a factor $4x$ in increased of the current flow for a larger unit cell detecting light at 4 µm over a unit cell operating at 1.5 µm.

- Lastly, the resistance difference between our device and the previous report is a factor $5x$. As photovoltages generated by the Seebeck effect are linked to thermoelectric currents across a PD by Ohms law the resistance has a direct influence on the current responsivity. This effect is seen also in Supplementary Fig. 15; The voltage responsivity divided by the current responsivity leads to ~3000 Ω matching the device resistance in the operation point.

Multiplying all estimated contributions leads to a total of

$$(Seebeck\ coeff.) \times (Absorption\ enhancement) \times (wavelength\ unit\ cell\ change)\ \times\ (resistance\ factor)$$

$$3\ x\ 4\ x\ 4\ x\ 5 \sim 10^2$$

explaining the two order of magnitude lower current responsivity in our devices. At the same time, this also shows the unmet potential of this architecture – improving the graphene quality, moving to multilayer graphene and changing the resonator design could lead to responsivities close to, or even higher than 0.1 A/W. Furthermore, the responsivity can be increased by etching the graphene channel[5]. This would also increase the resistance and as a result could further reduce thermal noise and improve the NEP. However, for high-speed operation, it would cause a larger mismatch between the typically 50 Ω terminated RF-circuits and would lead to worse power transmission. Employing multilayer graphene would improve the current responsivity due to higher absorption and lower resulting resistance. Furthermore, saturation could potentially be increased. However, multilayer graphene will also have a lower mobility which in turn leads to a reduced Seebeck coefficient and therefore lower responsivity. The responsivity and detectivity will therefore improve up to a certain number of layers. Supplementary Fig. 16 provides simulated absorption spectra for different graphene layer thicknesses. In addition to the total absorption of the metamaterial layer stack, the absorption per medium is also shown. A clear boost in absorption in graphene is observed for an increasing amount of graphene layers.

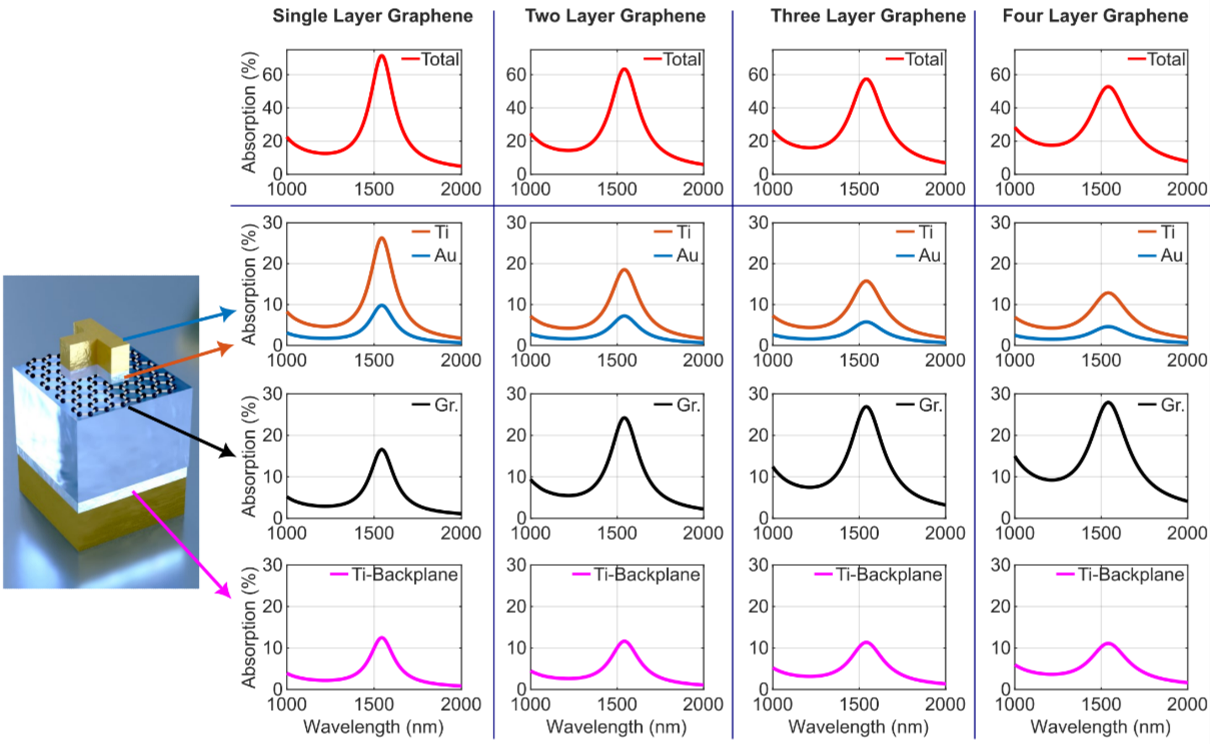

Supplementary Fig. 16: **Absorption per material of the architecture.**
Simulated absorption per material (rows) of the metamaterial absorber structure for different graphene thicknesses (columns). The first row shows the total absorption of the layer stack. The second row shows the absorption in the resonators consisting of gold and titanium. The third row shows the absorption in graphene which is strongly increasing with additional layers. Lastly, the fourth row shows the losses in the adhesion layer between the gold backplane and the alumina spacer layer.

Additionally, an overview of the state of the art of different graphene based PTE photodetectors is provided in the Supplementary Table 1. The table lists the graphene fabrication technology (exfoliated vs. photonic integrated circuit), the operation wavelength, the voltage responsivity, the maximum reported photovoltage and the bandwidth.

Supplementary Table 1: **Comparison of graphene based PTE photodetectors sorted by publication year.**
"Fab" stands for the fabrication method of the graphene sheet (Ex. - mechanical exfoliation, CVD - chemical vapor deposition). "Int." stands for integration scheme (PIC – photonic integrated circuit, FS – free space illuminated). λ is the illumination wavelength in nanometer. $\mathcal{R}_V$ is the reported voltage responsivity. V_{max} is the maximum reported photovoltage signal. The last column summarizes the reported bandwidths (BW) of the devices. The larger than ">" symbol denotes setup limited bandwidths.

Ref.	Year	Fab	Int.	$\lambda\ (nm)$	$\mathcal{R}_V$	V_{max}	BW
/10.1038/nnano.2014.182	2014	Ex.	FS	100'000	715 V/W	0.0027 mV	3.2 GHz
/10.1021/acs.nanolett.6b03374	2016	Ex.	PIC	1550	3.5 V/W	---	65 GHz
/10.1021/acsphotonics.8b01128	2018	Ex.	PIC	1550	4.7 V/W	1.5 mV<br>no power sweep	>18 GHz
/10.1021/acs.nanolett.9b02238	2019	CVD	PIC	1550	12.2 V/W	2.9 mV	42 GHz
/10.1038/s41467-020-20115-1	2020	Ex.	FS	4000	27 V/W	0.35 mV	>4 kHz
/10.1021/acsnano.0c02738	2020	CVD, monocryst.	PIC	1550	6 V/W	4 mV<br>no power sweep	>67 GHz
/10.1038/s41467-021-21137-z	2021	CVD, monocryst.	PIC	1550	3.5 V/W	15.1 mV<br>loss of linearity	70 GHz
/10.1038/s41467-021-23436-x	2021	Ex.	PIC	1550	90 V/W	38.6 mV<br>loss of linearity	12 GHz
/10.1038/s41566-021-00819-6	2021	Ex.	FS	4000	15.6 V/W	6.8 mV	0.52 MHz
/10.1038/s41467-022-31607-7	2022	CVD	PIC	5200	1.5 V/W	0.015 mV<br>no power sweep	> 1 MHz
/10.1038/s41566-022-01115-7	2023	Ex.	FS	4000	392 V/W	3 mV	> 0.39 MHz
This work	2024	CVD	FS	1550	1.6 V/W	18.5 mV<br>loss of linearity	420 GHz

Supplementary Note 9: Discussion on Frequency Response and RC cut-off

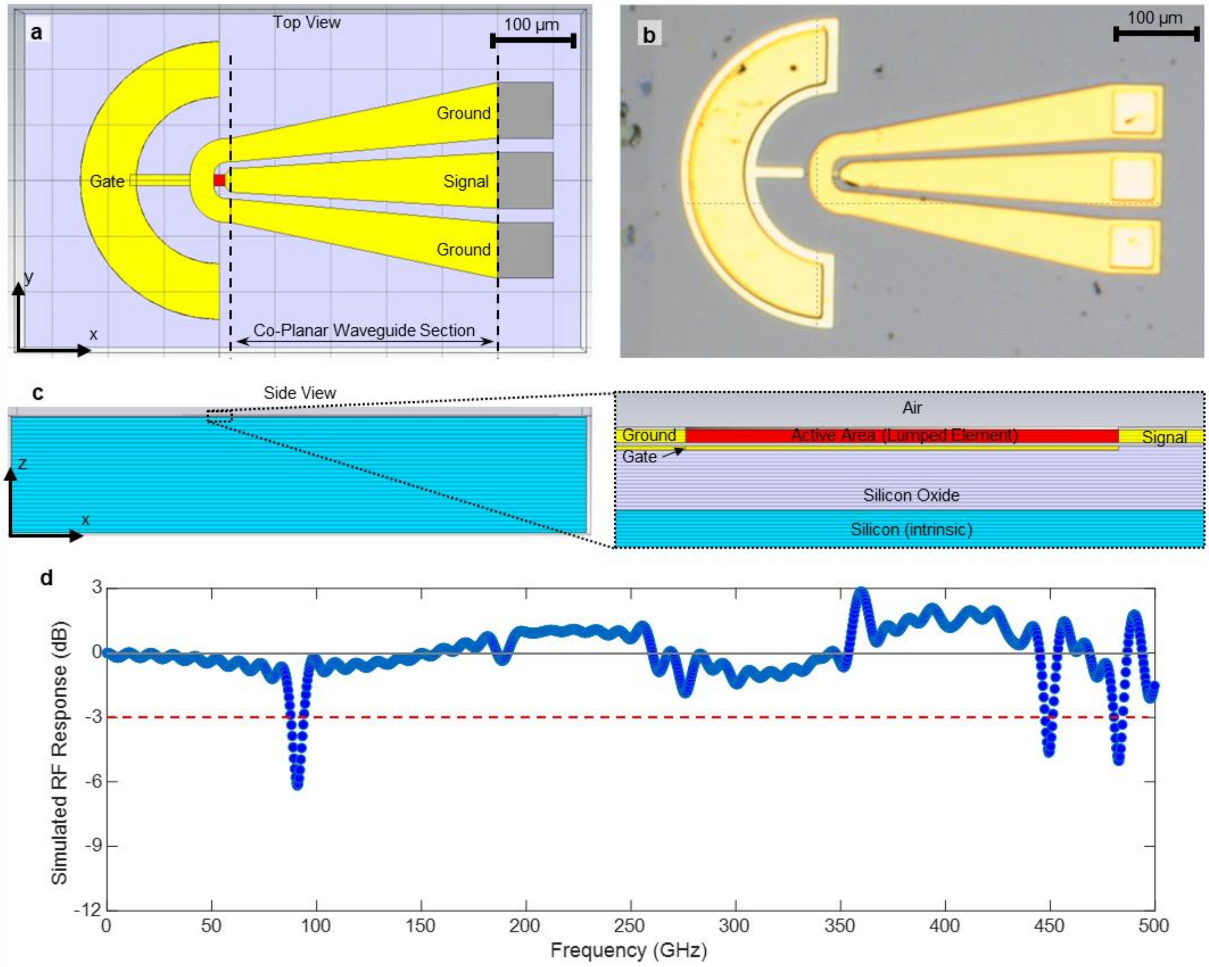

Supplementary Fig. 17 **Radio frequency pad design.**
a Top view of the full device geometry implemented in the simulation environment (CST Studio). The contact pads (gray squares) are connected to an optimized co-planar waveguide section. The active area of the device is represented in red, which is implemented as a lumped element. **b** Corresponding optical microscope image of a fabricated device. **c** Side view of the layer stack within the simulation environment. The zoom-in shows the active region layer stack. The substrate is silicon (intrinsic) with a 1µm thick thermally grown silicon oxide layer. Within the silicon oxide the gold gate is buried. A layer of aluminum oxide is on top followed by the active area of the device, and the ground and signal lines. A 50 nm aluminum oxide passivation layer is added on top. The structure is surrounded by air. **d** Simulated RF response from 0 to 500 GHz for the above depicted geometry. No roll-off behaviour is found.

To ensure ideal operation at high frequencies we optimized the contact pad design. Supplementary Fig. 17 illustrates the design. We implement the structure in CST Studio and perform time domain simulations. The RF pad design is illustrated in Supplementary Fig. 17a. Three contact pads (gray squares) modelled as perfect electric conductors are connected to the co-planar waveguides (CPW) forming the Ground-Signal-Ground (GSG) structure. The CPW was optimized in a first step to match the wave impedance of the contact structure to the 50 Ω of the RF circuit. For this, only the CPW structure connected terminated with two ports have been simulated separately. The active area of the device was fixed at 10 µm as well as the pitch of the pads which was set to 50 µm. Therefore, the parameters to optimize were the widths of the metal lines, the gaps between the lines and the length of the CPW. These parameters were optimized until the line impedance was close to 50 Ω. We note that the material layer stack illustrated in Supplementary Fig. 17c is determined by the metamaterial design as illustrated in Supplementary Fig. 8.

Using the optimized CPW geometry we simulated the full device as illustrated in Supplementary Fig. 17a. The active area of the device is here simply approximated as a lumped element with a resistance of 6000 Ω. At the same spot a port for the excitation signal is implemented mimicking the photoresponse of the PD. The transmitted and received signal is then detected at the gray GSG pads which are terminated with 50 Ω. The resulting simulated RF response is shown in Supplementary Fig. 17d. No roll-off behaviour is found in the range from 0 to 500 GHz. Oscillation in the spectrum stem from small impedance mismatches between the active area and the probing. Strong drops in the

frequency range have been identified as resonant coupling to the gate pad. In the measurements we did not experience these sharp drops which we assume stems from damping in the gate due to resistive losses.

To estimate the cut-off frequency of the bolometric (BOL) effect we extract the top left corner points from Figures 5c-h of the main text. At this operation point we have the strongest BOL response and are furthest away from the other effects. These data points are shown in Supplementary Fig. 18. Assuming the BOL effect has the same roll-off behaviour as the PTE-DC (i.e., 60 dB/dec), we estimate from these data points a 3 dB cut-off frequency between 250 and 330 GHz.

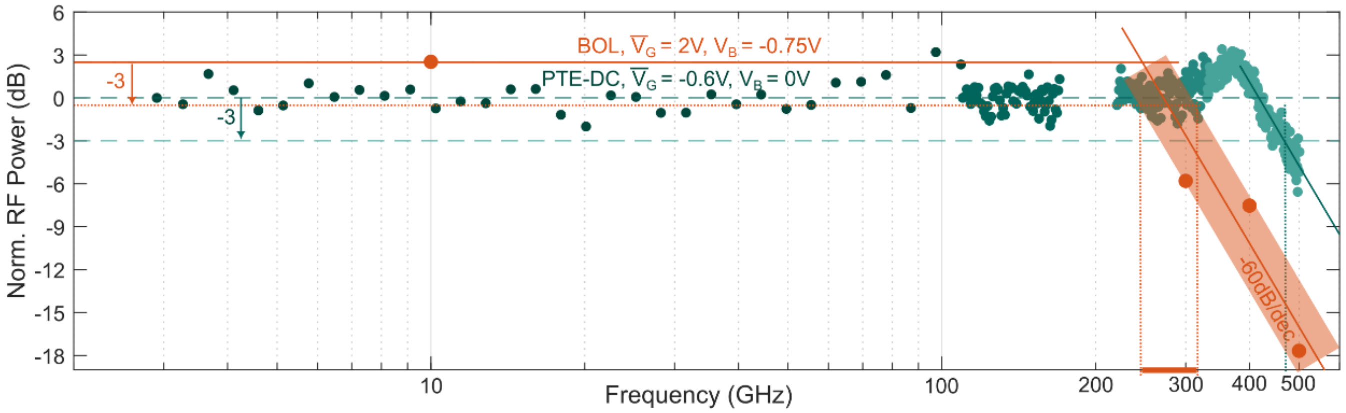

Supplementary Fig. 18: **3 dB cut-off frequency estimation of the bolometric effect.**
Green point correspond to photothermoelectric induced directional currents (PTE-DC) with 0 V bias and -0.6 V gate voltage with respect to the Dirac point position (corresponding to the measurement shown in Fig. 5). A roll-off with -60 dB/dec is found. Orange points correspond to bolometric (BOL) operation with -0.75 V bias and 2 V gate voltage with respect to the Dirac point position. Assuming the same roll-off slope a 3 dB cut-off frequency between 250 and 330 GHz is estimated.

SUPPLEMENTARY REFERENCES

1. Muench, J. E. *et al.* Waveguide-Integrated, Plasmonic Enhanced Graphene Photodetectors. *Nano Lett.* **19**, 7632–7644 (2019).
2. Gungor, A. C. *et al.* Modeling Hydrodynamic Charge Transport in Graphene. *Materials* **15**, 4141 (2022).
3. Mišeikis, V. *et al.* Ultrafast, Zero-Bias, Graphene Photodetectors with Polymeric Gate Dielectric on Passive Photonic Waveguides. *ACS Nano* **14**, 11190–11204 (2020).
4. Wei, J., Xu, C., Dong, B., Qiu, C.-W. & Lee, C. Mid-infrared semimetal polarization detectors with configurable polarity transition. *Nat. Photon.* **15**, 614–621 (2021).
5. Wei, J. *et al.* Geometric filterless photodetectors for mid-infrared spin light. *Nat. Photon.* 1–8 (2022) doi:10.1038/s41566-022-01115-7.
6. Levitov, L. & Falkovich, G. Electron viscosity, current vortices and negative nonlocal resistance in graphene. *Nature Phys* **12**, 672–676 (2016).
7. Wei, J. *et al.* Zero-bias mid-infrared graphene photodetectors with bulk photoresponse and calibration-free polarization detection. *Nat Commun* **11**, 6404 (2020).
8. Martin, J. *et al.* Observation of electron–hole puddles in graphene using a scanning single-electron transistor. *Nature Phys* **4**, 144–148 (2008).
